# Bright Molecular Strain Probe Templates for Reporting Protein–Protein Interactions

**DOI:** 10.3390/s23073498

**Published:** 2023-03-27

**Authors:** Sung-Bae Kim, Tadaomi Furuta, Genta Kamiya, Nobuo Kitada, Ramasamy Paulmurugan, Shojiro A. Maki

**Affiliations:** 1 Environmental Management Research Institute (EMRI), National Institute of Advanced Industrial Science and Technology (AIST), Tsukuba 305-8569, Japan; 2 School of Life Science and Technology, Tokyo Institute of Technology, Yokohama 226-8501, Japan; 3 Department of Engineering Science, Graduate School of Informatics and Engineering, The University of Electro-Communications, Chofu 182-8585, Japan; 4 Molecular Imaging Program at Stanford, Bio-X Program, Stanford University School of Medicine, Palo Alto, CA 94304, USA

**Keywords:** molecular strain probe, protein–protein interactions, *Renilla* luciferase, artificial luciferase, bioluminescence, template

## Abstract

Imaging protein–protein interactions (PPIs) is a hot topic in molecular medicine in the postgenomic sequencing era. In the present study, we report bright and highly sensitive single-chain molecular strain probe templates which embed full-length *Renilla* luciferase 8.6-535SG (RLuc86SG) or Artificial luciferase 49 (ALuc49) as reporters. These reporters were deployed between FKBP-rapamycin binding domain (FRB) and FK506-binding protein (FKBP) as a PPI model. This unique molecular design was conceptualized to exploit molecular strains of the sandwiched reporters appended by rapamycin-triggered intramolecular PPIs. The ligand-sensing properties of the templates were maximized by interface truncations and substrate modulation. The highest fold intensities, 9.4 and 16.6, of the templates were accomplished with RLuc86SG and ALuc49, respectively. The spectra of the templates, according to substrates, revealed that the colors are tunable to blue, green, and yellow. The putative substrate-binding chemistry and the working mechanisms of the probes were computationally modeled in the presence or absence of rapamycin. Considering that the molecular strain probe templates are applicable to other PPI models, the present approach would broaden the scope of the bioassay toolbox, which harnesses the privilege of luciferase reporters and the unique concept of the molecular strain probes into bioassays and molecular imaging.

## 1. Introduction

Bioluminescence (BL) is an emerging optical readout that can be utilized in various bioassays and molecular imaging. BL is generated through oxidative catalyzation of a specific substrate called “luciferin” by an enzyme “luciferase” in the presence of molecular oxygen (O_2_) [1]. BL-based assay systems generally have a long linear dynamic range, low background signal with high signal-to-background (S/B) ratios, biocompatibility, simple molecular design, a versatile utility in bioassay systems, and low cost [2,3].

To date, many bioassay systems have capitalized on BL to determine molecular events in the context of live cells and animal models. For example, bioluminescence resonance energy transfer (BRET) is a popular modality to assay protein–protein interactions (PPIs) in cells and live animals. BRET is based on the nonradiative energy transfer from a luciferase as the energy donor, and a fluorescent protein as the energy acceptor [4,5]. However, BRET probes have the following negative qualities: (i) the risk of detecting nonspecific signals because of random interactions, (ii) the influence of slow maturation and narrow Stokes shift of the fluorescent protein, (iii) the characteristic of having poor signal-to-background (S/B) ratios through low spectral separation, and (iv) poor absolute intensities [6,7].

Protein-fragment complementation assays (PCAs) also provide a unique strategy to assay PPIs in biological systems [7]. A reporter enzyme in the PCA probe is dissected into N- and C-terminal fragments, and thus, loses its enzymatic activity temporarily. The N- and C-terminal fragments are genetically fused to a pair of target proteins. When the two target proteins are brought into proximity by the PPI, the reporter fragments are complemented together, and their enzymatic activities recover [8]. However, PCAs inevitably destroy the intrinsic enzymatic scaffold of the reporter protein, and generally it recovers merely 0.5–5% of the original enzyme activity [9,10]. Hence, this drawback results in poor signal outputs. In addition, this strategy requires a sophisticated probe design and a tedious optimization step in deciding the optimal dissection site in the enzyme sequence. If the probe design fails, steric constraints may impede the association of enzyme fragments inside the probe [7].

The most conventional bioassay systems for determining molecular events in cells may be the “reporter-gene assay” and “two-hybrid assay” systems [11,12]. These systems make use of an expression vector, encoding a specific promoter sequence linked to the coding region that regulates the transcription of the luciferase reporter. A ligand-activated transcription factor binds to the promoter sequence and triggers expression of the luciferase reporter accumulation in cells, which can be measured at various time points. However, these transcriptional assay systems include some drawbacks, as follows: (i) monitoring temporal dynamics of target proteins is limited because it requires an hours-long stimulation time for the reporter accumulation to be detected using the assay systems; (ii) the system requires nuclear trafficking of proteins of interest, because their proximity to the transcriptional machinery is needed for the expression of the reporter proteins; and (iii) these reporters can be expressed even in basal cell culture conditions without any stimulator, which can lead to a high background signal and elevates nonspecific false-positive responses [2].

To address the limitations of the conventional bioassays, Kim et al. previously developed a unique, nontranscriptional assay system indexing molecular strains of a luciferase artificially appended by PPIs [13,14]. The studies reported that an artificially appended molecular strain to a full-length luciferase greatly elevates its enzymatic activities by enhancing the substrate turnover rates. For the basic probe design, the full-length luciferase was placed between two component proteins of interest with minimal linker lengths. To date, Kim et al. have examined many beetle and marine luciferases, and found that *Renilla* luciferase 8 (RLuc8) and artificial luciferase 23 (ALuc23) were suitable for designing such molecular strain probes. In the probe design, the full-length RLuc8 was sandwiched between the ligand-binding domain of estrogen receptor α (ER LBD) and Src homology 2 (SH2) domain; this fusion protein enhanced the light intensities estrogen-dependently [13]. Similarly, the full-length ALuc23 without secretion peptide (SP) was deployed between the FKBP-rapamycin binding domain (FRB) and FK506-binding protein (FKBP), and showed an excellent ligand-sensing performance as a bioluminescent probe [14]. The imaging system even works in a cell-free condition [15], and in its application to a BRET system [5]. Although these molecular strain probes are conceptually unique and generalizable to any enzymes and PPI models, they still possess challenges which need improvement in ligand-sensing properties, including absolute signal intensities and S/B ratios.

In this study, we developed bright and sensitive single-chain molecular strain probe templates based on *Renilla* luciferase 8.6-535SG (RLuc86SG) and artificial luciferase 49 (ALuc49). These templates were designed to leverage the brightness of RLuc86SG and ALuc49, and the unique concept of molecular strain probes by deploying the luciferase between the FRB and FKBP as the model PPI. To improve the ligand-sensing properties of the templates, including S/B ratios, the templates were characterized by including: truncation of the interface regions, modulation of coelenterazine (CTZ) analogues, and optimization of ligand concentrations. These efforts allowed us to accomplish the highest fold intensities of approximately >10 times with the templates in the presence of native CTZ (nCTZ) or its analogues. The BL spectra of the templates were determined, and the colors were tunable to blue, green, and yellow using our precedent substrates. The putative substrate-binding chemistry and the working mechanisms of the templates were computationally modeled in the presence or absence of rapamycin.

The novelty of this study mainly lies in the following points: (i) we conceptualize unique single-chain molecular strain probes with new luciferases to determine PPIs through combining multiple control studies and computational modeling; (ii) we demonstrate highly bright single-chain molecular strain probes with more than 10-fold enhancement of S/B ratios for the first time; and (iii) we greatly expand the color variation of molecular strain probes from blue to yellow-orange with combination of the substrates, that were invented by the authors. Considering that the molecular strain probe templates are applicable to other PPI models, the approach may broaden the scope of the bioassay toolbox that harnesses the privilege of luciferase reporters and the unique concept of the molecular strain probes in bioassays and molecular imaging.

## 2. Materials and Methods

### 2.1. Reagents

The native CTZ (nCTZ) was purchased from Nanolight Technology (Pinetop, AZ, USA). DBlueC and Prolume Purple 2 were obtained from Biotium (Fremont, CA, USA) and Nanolight Technology, respectively. The other substrates, BBlue2.3 and 6etOH-CTZ, were obtained from our previous studies [4,16]. Similarly, we obtained the cDNAs of the following marine luciferases cloned in pcDNA3.1(+) vector backbone from our precedent studies, i.e., *Gaussia* luciferase (GLuc), *Renilla* luciferase (RLuc), *Renilla* luciferase 8 (RLuc8), *Renilla* luciferase 8.6-535SG (RLuc86SG), Artificial luciferase 16 (ALuc16, GenBank MF817967), Artificial luciferase 23 (ALuc23; GenBank MF817968), ALuc47 (GenBank MF817975), and ALuc49 (GenBank MF817976) [17,18].

The cDNAs encoding human FK506-binding protein (FKBP, 12 kDa, GenBank AAP36774.1) and human FKBP-rapamycin binding domain (FRB, 11 kDa, Protein Data Bank 1AUE A) were custom-synthesized by Eurofins Genomics (Tokyo, Japan) based on the sequence information of public databases, as described in our previous study [14].

### 2.2. Construction of Mammalian Expression Vectors Encoding Various Molecular Strain Probes

**Figure 1:**
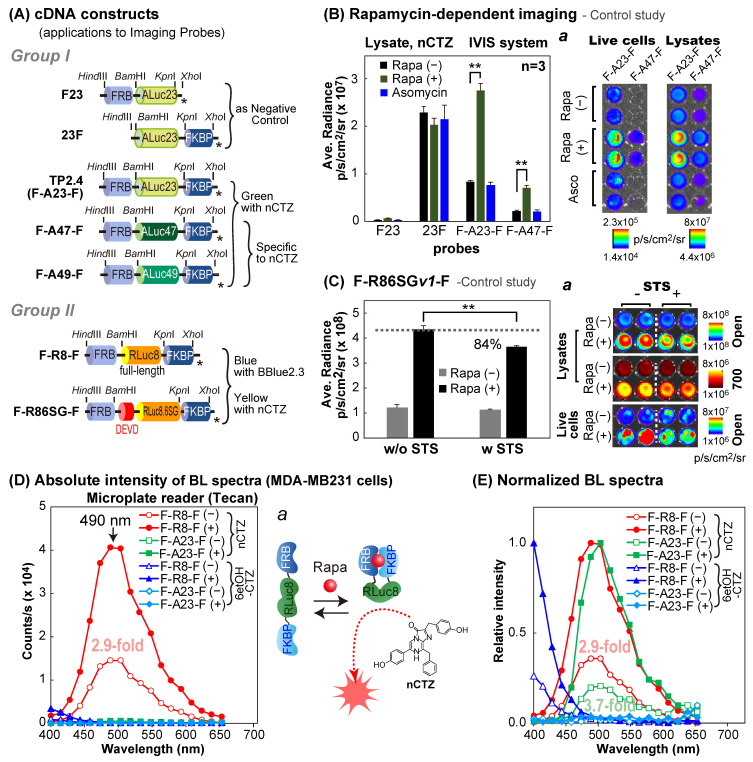
Schematic illustration of new molecular strain probes for imaging specific molecular events in the intracellular compartments of mammalian cells. (**A**) cDNA constructs of various BL imaging probes, which were categorized into two groups according to the molecular structures. DEVD means the Asp-Glu-Val-Asp tetrapeptide sequence as the substrate of caspases 3 and 7. The asterisks mean stop codons. (**B**) A negative control study to examine if BL intensities of the created molecular strain probes index the molecular strain of the probes. The probes F23 and 23F cannot append molecular strain to the sandwiched luciferase, ALuc23, whereas F23F and F47F enhance the BL intensities in a rapamycin-dependent manner. The *p*-value (Student’s *t*-test) is ** < 0.01. Inset *a* highlights the corresponding BL images that were enhanced after the addition of rapamycin. (**C**) A negative control study to examine if F-R86SG-F is truly activated by the molecular strain appended by the addition of rapamycin under the influence of STS. Inset *a* shows the corresponding BL images that were enhanced after the addition of rapamycin. The *p*-value (Student’s *t*-test) is ** < 0.01. (**D**) The absolute BL spectra of F-R8-F and F-A23-F in the presence (closed markers (+)) or absence (opened markers (−)) of rapamycin. The elevation in the BL intensities were determined after addition of nCTZ or 6etOH-CTZ. Inset *a* illustrates a putative working mechanism of F-R8-F after rapamycin addition. (**E**) The normalized BL spectra of F-R8-F and F-A23-F in the presence (closed markers (+)) or absence (opened markers (−)) of rapamycin.

**Figure 2:**
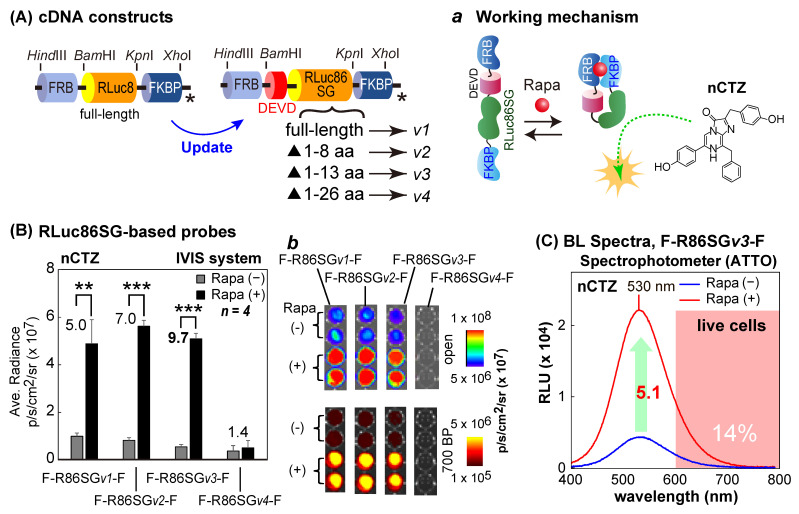
Characterization of the molecular strain probes based on RLuc86SG. (**A**) The cDNA constructs of new RLuc86SG-based molecular strain probes. Inset *a* illustrates the putative working mechanism of F-R86SG-F after rapamycin addition. The asterisks mean stop codons. (**B**) Rapamycin-driven optical intensities of the RLuc-based strain probes. The numbers on the bars indicate the fold intensities. The inset *b* shows the corresponding BL images before and after rapamycin stimulation. The *p*-values (Student’s *t*-test) are ** < 0.01 and *** < 0.001. (**C**) Representative BL spectra of F-R86SG*v3*-F before and after rapamycin stimulation. The red shadow marks the red and NIR region longer than 600 nm. Abbreviations: Rapa, rapamycin; RLU, relative light unit.

**Figure 3:**
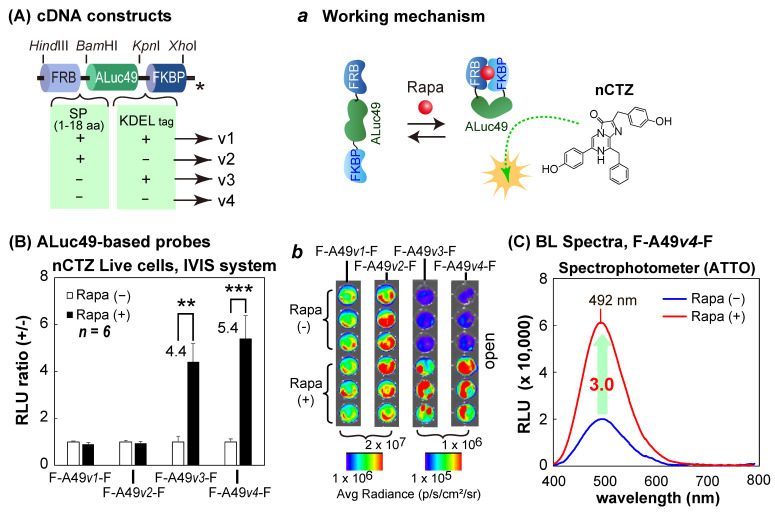
Characterization of the molecular strain probes based on ALuc49. (**A**) The cDNA constructs of ALuc49-based molecular strain probes. The asterisks mean stop codons. The inset *a* illustrates the working mechanism of F-A49-F in the presence of rapamycin (Rapa). (**B**) Rapamycin-driven optical intensities of the ALuc-based strain probes. The numbers on the bars indicate the fold intensities. The inset *b* shows the corresponding BL images before and after rapamycin stimulation. The *p*-values (Student’s *t*-test) are ** < 0.01 and *** < 0.001. (**C**) The BL spectra of F-A49*v4*-F in the presence or absence of rapamycin.

A series of cDNA constructs encoding the present molecular strain probes were made through a tandem linkage of cDNA blocks encoding FRB, marine luciferases, and FKBP, as shown in Figure 1A, Figure 2A and Figure 3A. In particular, the cDNA blocks were generated by polymerase chain reaction (PCR) using corresponding primers introduced with unique restriction sites, *Hind*III/*Bam*HI (to FRB), *Bam*HI/*Kpn*I (to the luciferase), or *Kpn*I/*Xho*I (to FKBP) at the 5′ and 3′ ends, respectively. Each molecular strain probe was differentiated by inserting various cDNA fragments encoding the luciferases between the cDNA fragments of FRB and FKBP.

The first series of molecular strain probes was made by ligating one of the following cDNA fragments encoding various sizes of RLuc86SG into the restriction sites *Bam*HI/*Kpn*I between the cDNA fragments of FRB and FKBP: i.e., (i) full-length RLuc86SG, (ii) 1–8 AA-deleted RLuc86SG at the N-terminal, (iii) 1–13 AA-deleted RLuc86SG at the N-terminal, and (iv) 1–26 AA-deleted RLuc86SG at the N-terminal. The corresponding probes after expression were named F-R86SG*v1*-F, F-R86SG*v2*-F, F-R86SG*v3*-F, and F-R86SG*v4*-F, respectively (Figure 2A).

The second series of molecular strain probes was constructed by inserting one of the following cDNA fragments encoding ALuc49 into the restriction sites *Bam*HI/*Kpn*I between the cDNA fragments of FRB and FKBP: i.e., (i) ALuc49 with the innate secretion peptide (SP) and the ER-retention signal peptide (KDEL), (ii) ALuc49 with SP and without KDEL, (iii) ALuc49 without the SP and with KDEL, and (iv) ALuc49 without both the SP and KDEL. The corresponding probes after expression were named F-A49*v1*-F, F-A49*v2*-F, F-A49*v3*-F, and F-A49*v4*-F, respectively (Figure 3A).

In the strain probe design, the linker lengths connecting each cDNA fragment were minimized to efficiently append the molecular strain to the sandwiched luciferase inside the single-chain probe after expression. The cDNA blocks were digested by the corresponding restriction enzymes (New England Biolabs, Ipswich, MA, USA), ligated with a ligation kit (Takara Bio, Tokyo, Japan), and finally subcloned into pcDNA 3.1(+) vector (Invitrogen (Thermo Fisher Scientific, Waltham, MA, USA)) after ligation using the *Hind*III and *Xho*I sites.

As a negative control, we made a series of “control” plasmids of the strain probes, in which the cDNA construct is deficient of fragments encoding FRB at the N-terminal, or FKBP at the C-terminal, as shown in Figure 1A.

The sequential fidelity of the cDNA constructs was finally confirmed with a genetic sequence analyzer by order (Eurofin Genomics, Tokyo, Japan).

### 2.3. Negative Control Studies of the Molecular Strain Probes Based on Marine Luciferases

Two negative control studies were conducted to confirm the ligand specificity, as well as the intended BL intensities and the specificities of the molecular strain probes (Figure 1).

For the first negative control study (Figure 1B), MDA-MB231 cells were cultured in a 6-well microplate to approximately 70% confluency and transiently transfected with pcDNA3.1(+) vector encoding F23, 23F, F-A23-F, F-A47-F, or F-A49-F using a lipofection reagent (TransIT-LT1, Mirus (Madison, WI, USA)). After overnight incubation, the cells were harvested and subcultured in a 96-well black-frame microplate and further incubated in a 5% (*v*/*v*) CO_2_ incubator for 12 h. The culture media of the cells were then replaced with the same media containing vehicle (0.1% (*v*/*v*) DMSO), rapamycin (final concentration: 10^−7^ M), or ascomycin (final concentration: 10^−7^ M). The cells were further incubated overnight. The overnight incubation of the cells with rapamycin was performed because rapamycin is a chemically hydrophilic molecule, which requires extended incubation time to achieve a sufficient amount of intracellular rapamycin concentration in the cytosolic compartment to activate PPIs. The wells containing the cells were conceptionally divided into two sections. The culture media in both sections were completely removed and the cells in the first section were lysed with a lysis buffer (Promega, Madison, WI, USA), and the other section was sealed to prevent evaporation before live cell imaging. Then, the wells in both sections were injected with 40 μL of the PBS buffer containing nCTZ. The corresponding BL images were determined using the IVIS Spectrum imaging system (PerkinElmer, Waltham, MA, USA) and analyzed using the Living Image version 4.7 software. Optical filters were not applied for the measurement.

For the second negative control study (Figure 1C), COS-7 cells grown in a 6-well microplate to approximately 70% confluency were transiently transfected with pcDNA3.1(+) vector encoding F-R86SG*v1*-F. The cells were incubated for 12 h, harvested, and subcultured into a 96-well black-wall microplate. After an additional overnight incubation, the cells were stimulated overnight with 10^−7^ M rapamycin. The wells containing the cells were also conceptionally divided into two sections. The first section was stimulated with staurosporine (STS) dissolved in the culture media for 5 minutes, whereas the second section was stimulated with the vehicle (0.1%(*v*/*v*) DMSO). The culture media were completely removed. The cells in the wells in the half section were lysed and the others remained as is. The corresponding BL images were determined using the same method in Figure 1B.

### 2.4. Rapamycin-Driven Change in the Spectra of F-R8-F or F-A23-F

The change in the spectra of the cells containing F-R8-F or F-A23-F was determined in the presence or absence of rapamycin (Figure 1D,E).

MDA-MB231 cells grown in a 6-well microplate were transiently transfected with pcDNA3.1(+) vector encoding F-R8-F or F-A23-F and incubated further for 24 h. The cells were subcultured in a 96-well black-frame microplate (Nunc, Thermo Fisher Scientific, Waltham, MA, USA) and incubated overnight. Then, the cells were stimulated overnight with either 10^−7^ M rapamycin or its vehicle control (0.1% ethanol). The culture media were completely removed by aspiration, and the remaining cells were lysed by the addition of 40 μL of a lysis buffer (Promega) per well. After the injection of 40 μL of a PBS buffer containing nCTZ or 6etOH-CTZ (final concentration = 10 μg/mL), the BL spectra of each lysate in the microplate were determined immediately with a microplate reader equipped with a series of band-pass (BP) filters (Spark 10M, Tecan (Männedorf, Switzerland)) with varying optical filters from 398 to 653 nm, with 15 nm increments (each filter has a 15 nm BP window). The integration time for each measurement was one second.

### 2.5. Characterization of the Molecular Strain Probes Based on RLuc86SG and ALuc49

Because the molecular strain probe F-R86SG*v1*-F showed excellent sensitivity and specificity to ligand rapamycin in Figure 1C, the ligand sensitivity was characterized by truncating the N-terminal end of RLuc86SG inside the strain probe F-R86SG*v1*-F (Figure 2A,B).

First, COS-7 cells were cultured in a 6-well microplate until reaching 70% confluency. The cells were transiently transfected with pcDNA3.1(+) vector encoding F-R86SG*v1*-F, F-R86SG*v2*-F, F-R86SG*v3*-F, or F-R86SG*v4*-F using a lipofection reagent, TransIT-LT1 (Mirus), and incubated overnight. After overnight incubation, the cells were subcultured into a 96-well black-wall microplate. The cells were further incubated for 12 h. The cells were stimulated with either vehicle (0.1% (*v*/*v*) ethanol in the culture media) or 10^−7^ M rapamycin (final concentration) dissolved in the culture media and incubated overnight. The cells in the microplate were lysed by a lysis buffer (Promega) for 15 min and simultaneously injected with 50 μL of 100 μM nCTZ dissolved in PBS. The corresponding BL images were determined using the IVIS Spectrum optical imaging system with either an open or 700-nm BP filter. The obtained BL images were finally analyzed using the Living Image version 4.7 software.

The BL spectral variation of the cells containing F-R86SG*v3*-F was also determined in the presence or absence of rapamycin (Figure 2C). The cells were prepared and lysed using the same method in Figure 2B. Forty μL of the lysates was transferred to 200-μL PCR tubes, and further injected with 40 μL of the assay buffer (Promega) containing nCTZ. The corresponding BL spectra were determined with a high precision spectrophotometer (AB-1850, ATTO, Tokyo, Japan), which simultaneously acquires all the wavelengths and is not influenced by the optical decay by time. The spectral data were analyzed using the LumiFLSpectroCapture version 1.0 software (ATTO, Tokyo, Japan). In the spectra, RLU stands for relative light unit.

A new series of molecular strain probes, based on ALuc49, was further characterized, wherein four different versions of ALuc49 were sandwiched between the FRB and FKBP (Figure 3). The constructed probes were named as F-A49*v1*-F, F-A49*v2*-F, F-A49*v3*-F, and F-A49*v4*-F, as illustrated in Figure 3A. The probes were characterized with the same protocols in Figure 2B,C using COS-7 cells, and are summarized in Figure 3B,C.

We further highlighted the ligand-specific images of F-R86SG*v3*-F and F-A49*v4*-F in microslides (Supplementary Figure S4). COS-7 cells grown in a 6-well microplate were transiently transfected with pcDNA3.1(+) vector encoding F-R86SG*v3*-F or F-A49*v4*-F. The cells were incubated overnight, harvested, and subcultured into two 6-well microslides (µ-Slide VI 0.4, ibidi (Bayern, Germany)). The cells in the three left and right channels were stimulated with the culture medium containing vehicle (0.1% ethanol) and 10^−7^ M rapamycin (final concentration), respectively. After the complete aspiration of the stimulation media, the cells in the microslide were lysed using a Promega lysis buffer for 15 min, whereas those in the other microslide remained as is during the same time for live cell imaging. The channels were simultaneously injected with 50 μL of 100 μM nCTZ dissolved in PBS. The corresponding BL images were obtained using the IVIS Spectrum imaging system equipped with either an open or 700 nm BP filter. The obtained BL images were analyzed using the Living Image version 4.7 software.

### 2.6. Rapamycin and Substrate-Driven BL Spectra of F-R86SGv3-F and F-A49v4-F

**Figure 4:**
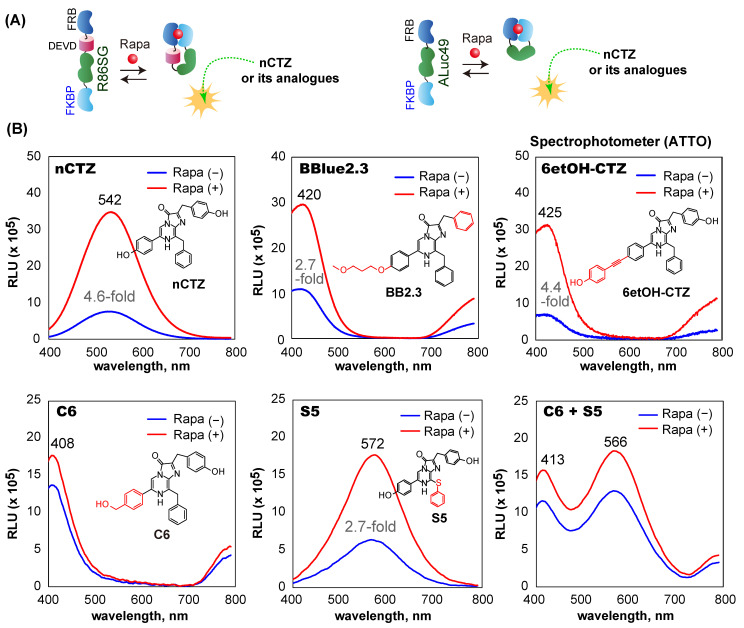
(**A**) The putative working mechanism of F-R86SG*v3*-F and F-A49*v4*-F in the presence of rapamycin. (**B**) BL spectra of cells coexpressing F-R86SG*v3*-F and F-A49*v4*-F according to various CTZ analogues. The BL spectra of COS-7 cells containing F-R86SG*v3*-F and F-A49*v4*-F were determined in the presence or absence of rapamycin. The chemical structures of the applied CTZ analogues were specified inside the graphs.

The variation of the BL spectra of the new single-chain molecular strain probes by rapamycin and the substrates was determined in living COS-7 cells (Figure 4). COS-7 cells were plated in 12-well microplates to a 70% confluency in 24 h. The cells were transiently cotransfected with F-R86SG*v3*-F and F-A49*v4*-F using a lipofection reagent, TransIT-LT1 (Mirus), according to the manufacturer’s instruction. The cells were incubated overnight in a 5% (*v*/*v*) CO_2_ incubator. The cells in each well were then stimulated with either vehicle (0.1% (*v*/*v*) ethanol) or 10^−7^ M rapamycin by replacing the culture media with a fresh medium containing the ligand. The cells were further incubated overnight, harvested through trypsinization, and collected by centrifugation. The cells were then resuspended in PBS at 1 × 10^5^ cells/mL. Forty μL of the cell suspension was mixed with 40 μL of the substrate solution containing nCTZ, BBlue2.3, 6etOH-CTZ, C6, S5, or the mixture of C6 and S5. The corresponding BL spectra were immediately determined with a high precision spectrophotometer (AB-1850). The spectral data were analyzed with the LumiFLSpectroCapture version 1.0 software.

### 2.7. Substrate-Driven Increase in Fold Intensities of the New Single-Chain Molecular Strain Probes

**Figure 5:**
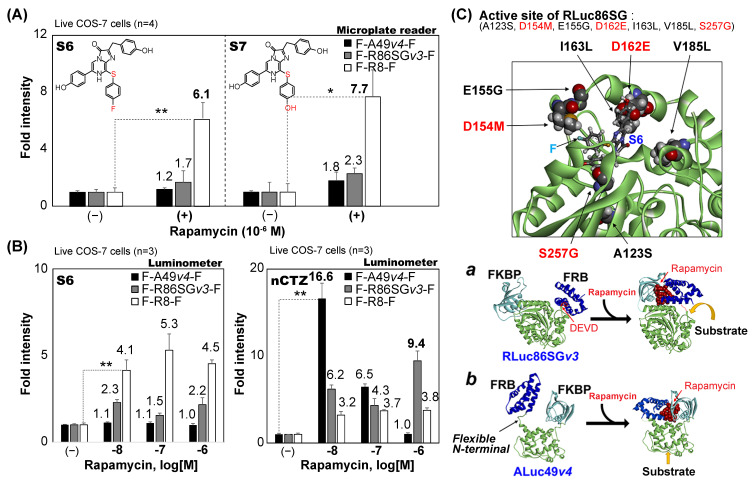
(**A**) Fold intensities of rapamycin-activated single-chain molecular strain probes, according to the substrates (S6 and S7). The *p*-values (Student’s *t*-test) are * < 0.1 and ** < 0.01. The data were obtained using a 96-well microplate reader. (**B**) Variation of the dose–responses of the single-chain molecular strain probes according to the substrates (S6 and nCTZ). The *p*-values (Student’s *t*-test) are ** < 0.01. The data were obtained using a luminometer. (**C**) A putative three-dimensional (3-D) structure of RLuc86SG bounded with S6, and working mechanisms of F-R86SG*v3*-F and F-A49*v4*-F. The structure of RLuc86SG with S6 was modeled according to a previous study [22], and the original substrate was manually modified to S6. The mutational residues (RLuc8 to RLuc86SG, 7 AA) and S6 are represented by spheres and stick, respectively. Insets *a* and *b* illustrate the working mechanisms of F-R86SG*v3*-F and F-A49*v4*-F, respectively (Experimental Procedures and Supplementary Information).

The increase in signal intensities of the new single-chain molecular strain probes, according to the substrates, was determined (Figure 5). For the experiment of Figure 5A, 1 × 10^4^ of COS-7 cells was seeded into each well of 96-well black-wall microplates. The cells were cultured overnight in the 5% (*v*/*v*) CO_2_ incubator. The cells were then transiently transfected with F-R8-F, F-R86SG*v3*-F, or F-A49*v4*-F, and further incubated overnight. The cells were then stimulated with either vehicle (0.1% ethanol) or 10^−7^ M rapamycin for six hours. The culture media in the microplate were aspirated and set in a microplate reader (TriStar3, Berthold, Germany). The corresponding BL intensities were determined after programmed automatic injection of 40 μL of the substrate solutions containing S6 or S7. The data were saved into an Excel file and analyzed with Excel 365 software (Microsoft, Redmond, WA, USA). 

For the experiment of Figure 5B, COS-7 cells were grown in 12-well microplates until the cell population reached 70% confluency. The cells were transiently transfected with F-R8-F, F-R86SG*v3*-F, or F-A49*v4*-F using the lipofection reagent TransIT-LT1 (Mirus). The cells were then incubated for 1day and harvested by trypsinization, followed by centrifugation. The cells were then resuspended in PBS at 5 × 10^5^ cells/mL. Twenty μL of the cell suspension was mixed with 30 μL of the substrate solution, dissolving either S6 or nCTZ in a 1.5 mL PCR tube. The corresponding BL intensities were determined using a luminometer (GloMax 20/20n, Promega).

### 2.8. Structural Modeling of Rluc86SG with S6 and Schematic Illustration of the Working Mechanism of F-R86SGv3-F and F-Aluc49v4-F

The putative 3D structure of RLuc86SG with S6 was created based on the X-ray crystal structure of RLuc8 (Figure 5C). The RLuc8 structure was obtained from the protein data bank (PDB: 7OMR [19]). Then, we introduced the seven residues (i.e., A123, D154, E155, D162, I163, V185, and S257) mutated to form RLuc86SG. Finally, the bound coelenteramide was manually modified to form the structure of substrate S6. The structural modeling was performed by Discovery Studio 2017 R2 (Dassault Systèmes, Vélizy-Villacoublay, France).

To illustrate a putative working mechanism of F-R86SG*v3*-F triggered by rapamycin, information on the RLuc8 and FKBP-FRB (with rapamycin) structures was first obtained from the protein data bank (PDB: 1FAP [20] and 7OMR [19]; the latter was modified to form RLuc86SG*v3*). Then, these components were manually deployed in the FRB-R86SG*v3*-FKBP order (in the presence (+) and absence (−) of rapamycin) (Figure 5C inset *a* and Supplementary Figure S2). In the FKBP–FRB-bound state, the substrate is accessible to the sandwiched R86SG*v3* without the steric hindrance of FRB.

Similarly, to illustrate a putative BL mechanism of F-A49*v4*-F, the ALuc49 structure was created beforehand by ColabFold [21] (then, the SP region at the N-terminal was deleted to feature ALuc49*v4*), and the FKBP-FRB structure was set with the same as used above. Then, the components were manually deployed in the FRB-ALuc49*v4*-FKBP in order (in the two states) (Figure 5C inset *b* and Supplementary Figure S3).

## 3. Results and Discussion

### 3.1. RLuc8, RLuc86SG, ALuc47, and ALuc49 Exert Innate, Distinctive Luciferin Specificity and Brightness

We first determined the BL intensities of selected marine luciferases stably expressed in live MDA-MB231 cells, according to the substrates (Supplementary Figure S1). The results showed that the strongest BL intensities were observed in the order of ALuc16, ALuc49, and RLuc86SG, followed by ALuc47 in the presence of substrate nCTZ (Supplementary Figure S1A). The average radiances and luciferin specificities of RLuc86SG and ALuc49 were determined, and these luciferases were found as bright as 1.6 × 10^7^ and 2.6 × 10^7^ p/s/cm^2^/sr, respectively, when they react with the substrate nCTZ. RLuc86SG showed considerable reactivity with the BBlue2.3 substrate, whereas ALuc49 did not. The weakest BL intensities of the marine luciferases were observed as low as 9.0 × 10^5^ p/s/cm^2^/sr when we used 6etOH-CTZ as substrate. This dramatic change in brightness among marine luciferases may be explained by the rigidity of the functional group at the C-6 position of the CTZ analogues: i.e., BBlue2.3, which carries a flexible allyl group at the C-6 position, whereas 6etOH-CTZ contains a rigid ethynyl group potentially causing steric hindrance inside the active sites of the luciferases.

We further determined the substrate specificity of RLuc8 and ALuc49 stably expressed in MDA-MB231 cells (Supplementary Figure S1B). The stable cells were established by G418 selection after transient transfection of the plasmids. The results showed that RLuc8 stably expressed in MDA-MB231 cells exhibited the brightest signal when reacted with nCTZ (5.1 × 10^7^ p/s/cm^2^/sr), followed by Prolume Purple 2 and BBlue2.3 substrates. In contrast, ALuc49 stably expressed in MDA-MB231 cells was highly specific only to nCTZ, and did not show significant reactivity with the other substrates.

### 3.2. The Negative Control Studies Reveal That Molecular Strain Probes Index the Intramolecular Strain Appended by PPIs

The present probes were conceptualized to exploit molecular strains of the sandwiched reporters appended by intramolecular PPIs. Because the concept of “molecular strain probe” needs to be proven with experimental evidence, two negative control studies were performed to confirm whether the proposed working mechanism is plausible: i.e., (i) rapamycin triggers interaction between FRB and FRBP and appends the molecular strain to the sandwiched full-length luciferase, and (ii) the sandwiched luciferase enhances the enzymatic activities in a ligand-dependent manner.

In the first negative control study, four different probes were designed to prove if the ligand-triggered intramolecular strain modulates the enzymatic activities: i.e., FRB-ALuc23 (F23) was deficient of FKBP, whereas ALuc23-FKBP (23F) had no FRB. Conversely, FRB-ALuc23-FKBP (F-A23-F) and FRB-ALuc47-FKBP (F-A47-F) carried both FRB and FKBP, which exert intramolecular PPIs (Figure 1A). The results showed that F23 exhibited weak absolute intensities as low as 6 × 10^4^ p/s/cm^2^/sr, and 23F failed to elevate the BL intensities after the addition of rapamycin as the agonist, or ascomycin as the antagonist (Figure 1B). It is characteristic that 23F exhibits strong background BL intensities even without the ligand. However, the F-A23-F and F-A47-F showed lower background BL intensities and successfully enhanced BL intensities with the addition of the agonist rapamycin, but not with the antagonist ascomycin. These rapamycin-driven features correspond with the results of our previous study, wherein F-A23-F reacted with our new substrates, namely, S5, S6, and C6 [22]. These results conclude the following: (i) FRB and FKBP are the essential components for appending the molecular strain to the sandwiched luciferases in the molecular strain probes, and (ii) modification of the N-terminal end of ALuc23 with a protein works in a way that suppresses the background intensities in the absence of rapamycin, after comparing the absolute intensities of F23, 23F, and F-A23-F.

In the second negative control study (Figure 1C), a new molecular strain probe was created to deploy RLuc86SG between FRB and FKBP (i.e., F-R86SG-F), and the modified N-terminal end of RLuc86SG was capped with an Asp-Glu-Val-Asp (DEVD) peptide for measuring apoptosis activities. This probe was designed to be fragmented after the stimulation of apoptosis in cells. It is because the substrate peptide (DEVD) inside the probe is the prototypical substrate sequence of caspases 3 and 7, and thus is immediately fragmented after apoptosis initiation [23]. The fragmented probes cannot append the molecular strain to the sandwiched luciferase, and show lower signals as a measure of apoptosis.

The absolute BL intensities of rapamycin- or vehicle-activated F-R86SG*v1*-F were determined in the presence or absence of STS as an apoptosis inducer (Figure 1C). The results showed that the absolute intensities of the reference side (rapamycin-free) were not changed in the presence or absence of STS, but those of the acting side (rapamycin) were significantly decreased (~84%) only in the case of STS stimulation. These results are interpreted as follows: (i) STS initiated the apoptosis through the activation of caspases, which cleaved the DEVD site inside F-R86SG*v1*-F; and (ii) the dissection negatively impacted the luciferase intensity of the acting side (with rapamycin) rather than that of the background, because the intramolecular tension is eased by the dissection.

The above results support the function of the molecular strain probe and the proposed mechanism: i.e., the single-chain molecular strain probe, FRB-Luciferase-FKBP, remain relaxed in the absence of rapamycin. The FRB and FKBP may sterically hinder the access of the substrates to the active sites of the sandwiched luciferase. The luciferase activity before exposure to rapamycin is relatively low. A dynamic conversance happens after rapamycin addition. Rapamycin triggers the intramolecular FRB–FKBP interactions, which appends the molecular strain to the sandwiched luciferases in the probes. The molecular strain may work in a way to expose or enlarge the active site to the aqueous phase containing the substrate, facilitating the access of the substrate in the aqueous phase. The enhanced luciferase activities lead to enhancement in the BL intensity.

### 3.3. RLuc86SG-Based Molecular Strain Probes Show Improved Fold BL Intensities While Retaining Absolute Signal Intensities

Because F-R8-F and F-A23-F showed successful ligand-sensing properties in response to rapamycin in the previous sections, the probe designs were optimized to improve the optical properties (Figure 2 and Figure 3). In the new molecular design, RLuc8 in F-R8-F was substituted with RLuc86SG (Figure 2), because RLuc86SG exhibited the highest BL intensity among RLuc variants and the unique substrate specificity in Supplementary Figure S1. In addition, the N-terminal end of RLuc86SG was truncated to optimize the linker length under the premise that the intramolecular strain is dominated by the tightness of the linker region. Among the four different versions constructed, F-R86SG*v3*-F showed the highest S/B ratio, 9.7-fold (530 nm), followed by 7.0-fold by F-R86SG*v2*-F, and 5.0-fold by F-R86SG*v1*-F. Whereas, F-R86SG*v4*-F completely lost its BL intensity and exerted low elevation (1.4-fold) in the BL signal in the presence of rapamycin. The putative BL mechanism of F-R86SG-F triggered by rapamycin is illustrated in Supplementary Figure S2A, which was similarly featured in our previous study [22]. In regard to the BL intensity of F-R86SG*v4*-F (Supplementary Figure S2B), the truncated region (1–26 AA) comprises the core β sheet of the enzyme, likely leading to misfolding or attenuation of BL.

Conventionally, when one improves the S/B ratios of probes, the absolute intensities are prone to be compromised: a typical example is the case of the TP3 series probe in our previous study [14]. However, the present study shows that the absolute intensities were almost sustained, and the S/B ratios were interestingly improved because of the decreased background intensities.

We previously suggested to categorize the bioassay systems based on transcriptional and nontranscriptional assay systems [2]. A reporter-gene assay and a two-hybrid assay were grouped in a family of transcriptional assay systems. However, in nontranscriptional assay systems, the probes are expressed beforehand, and prelocalized at the adequate level in intracellular compartments of interest. These types of probes are ready to develop BL upon stimulation of a signal. Therefore, it is explained that the present molecular strain probe systems intrinsically respond quickly to signals, and are generally expected to have higher S/N ratios than transcriptional assay systems.

### 3.4. F-R86SGv3-F and F-A49v4-F Show Diverse BL Spectra Based on the Substrates

The BL spectra of F-R86SG*v3*-F and F-A49*v4*-F were investigated in the presence or absence of rapamycin (Figure 4). F-R86SG*v3*-F and F-A49*v4*-F cotransfected in COS-7 cells dramatically changed the BL spectra by the addition of the ligand and the substrates. The cells showed the strongest BL spectrum peak at 542 nm when reacted with nCTZ (yellowish-green). The spectral intensities were approximately 4.6-fold enhanced upon exposure to rapamycin compared with the vehicle as the negative control. The BL spectral peaks with BBlue2.3, 6etOH-CTZ, and C6 exhibited a blue color at 420 nm, 425 nm, and 408 nm, respectively. The peaks were blue-shifted up to ca. 134 nm, compared with those with nCTZ. On the other hand, the cells showed a 30 nm red-shifted BL spectrum which peaked at 572 nm (yellow) with S5. The cells showed two spectral peaks at 413 and 566 nm (violet and yellow) when their BL was developed with a 50:50 mixture of C6 and S5. The BL spectral shapes and peaks are unique when used as the signature of the probes, enabling their rapid recognition in multiplex systems.

The above unique spectral peaks indicate that the BL spectra are mostly contributed by F-R86SG*v3*-F (530 nm; Figure 2C) rather than F-A49*v4*-F (492 nm, Figure 3C), because the characteristic peak of F-A49*v4*-F at 492 nm was not observed in the spectra in Figure 4B. In addition, the blue- and red-shifted BL peaks near 410 and 570 nm correspond with the typical peaks of RLuc86SG in our previous studies [22,24]. To investigate why the contributed peak of F-A49*v4*-F is not observed in the spectra, we compared the absolute intensities of F-R86SG*v3*-F and F-A49*v4*-F (Supplementary Figure S5). The results show that the live cells containing F-R86SG*v3*-F emit 99.7- and 114.1-fold stronger BL intensities than those containing F-A49*v4*-F in the presence of BBlue2.3 and nCTZ, respectively. The average radiances of the cells containing F-R86SG*v3*-F and F-A49*v3*-F were 9.7 × 10^7^ and 6.7 × 10^5^ p/s/cm²/sr, respectively. The superior BL intensities of F-R86SG*v3*-F to F-A49*v3*-F do not correspond with the results in Supplementary Figure S1A, reporting that ALuc49 is brighter than RLuc86SG. Supplementary Figures S1 and S5 also reveal that F-A23-F is much weaker than the net ALuc23 in the absolute BL intensities.

These results are interpreted as follows: (i) ALucs, such as ALuc23 and ALuc49, significantly reduce their BL intensities after the N- and C-terminal ends are modified with FRB and FKBP, whereas RLuc86SG retains the original BL intensities even after the modification of the N- and C-terminal ends, (ii) the great gap in the absolute intensities between F-R86SG*v3*-F and F-A49*v3*-F proves why the BL spectra of those coexpressions are mostly contributed by R86SGv3-F (Figure 4), and (iii) the peak positions (colors) and intensities of the spectra are dominated by the substrates.

### 3.5. The Fold Intensities of F-R86SG-F and F-A49-F Are Driven by the Applied Substrates

Single-chain molecular strain probes were originally characterized using nCTZ as substrate, as shown in Figure 1, Figure 2 and Figure 3. However, the spectra in Figure 4 indicated that the applied substrates drive the fold intensities of the molecular strain probes. Therefore, we carefully investigated the fold intensities of the molecular strain probes in detail, according to the substrates, in the presence or absence of rapamycin (Figure 5). The highest fold intensities of 6.1 and 7.7 were observed with a microplate reader when F-R8-F reacted with S6 and S7, respectively. In contrast, F-A49*v4*-F and F-R86SG*v3*-F showed relatively low fold intensities from 1.2 to 2.3 when reacted with the same S6 and S7 as substrate, respectively (Figure 5A).

The fold intensities at varying concentrations of rapamycin were further determined in the presence of S6 or nCTZ using a luminometer assay (Figure 5B). The results, when reacted with S6, showed that F-R8-F keeps the highest fold intensities, ranging from 4.1- to 5.3-fold, compared with F-A49*v4*-F and F-R86SG*v3*-F, despite the varying concentrations of rapamycin. However, this feature was completely reversed when the fold intensities were determined using nCTZ. The highest fold intensities, of 16.6, were obtained when F-A49*v4*-F reacts with nCTZ after stimulation with 10^−8^ M rapamycin. This highest fold intensity of F-A49*v4*-F was reduced to 6.5- and 1.0-fold by increasing the concentrations of rapamycin to 10^−7^ M and 10^−6^ M, respectively. In contrast, F-R86SG*v3*-F and F-R8-F did not show dramatic elevations in the fold intensities by increasing the rapamycin concentrations. The highest fold intensities of F-R86SG*v3*-F were determined with 10^−6^ M rapamycin in the presence of nCTZ. The fold intensities were reduced to 6.2- and 4.3-fold by decreasing the rapamycin concentration. The fold intensities of F-R8-F were relatively stable to around 3.7 in the tested rapamycin, with the concentrations ranging from 10^−8^ to 10^−6^ M. The corresponding kinetic and binding chemistry of rapamycin-triggered FRB–FKBP interactions were well investigated in several previous studies [25,26].

The overall results may be summarized as follows: (i) rapamycin is the ligand that elevates the fold intensities of the single-chain molecular strain probes, (ii) the optimal rapamycin concentrations for the best fold intensities may be different according to molecular strain probes, and (iii) the three parameters driving the fold intensities are the substrate, the rapamycin concentration, and the type of luciferase embedded in the molecular strain probe.

### 3.6. Putative Structures and Working Mechanisms of the Molecular Strain Probes

Because the X-ray crystallographic information of RLuc8 was reported previously, the structure of RLuc8 with coelenteramide was modified to create the putative binding mode of RLuc86SG with S6 (Figure 5C).

It is known that RLuc86SG has seven additional mutations from RLuc8, that are A123S, D154M, E155G, D162E, I163L, V185L, and S257G. These seven mutations in RLuc86SG are considered to determine the substrate specificity favoring nCTZ, different from RLuc8 preferring S6. The structural difference between nCTZ and S6 is observed at the C-8 position: i.e., S6 features a fluorine (F) tail and a sulfur (S) elbow at the C-8 position of S6. The 3D structure in Figure 5C shows that the amino acids M154 and E162 can interact with the functional groups at the C-8 and C-6 positions of the S6 substrate, respectively, and G257 is close to its C-2 position, suggesting that they are the determinants of the substrate specificity of RLuc86SG, and it is different from RLuc8.

The insets *a* and *b* in Figure 5C illustrate the working mechanisms of F-R86SG*v3*-F and F-A49*v4*-F. Rapamycin triggers the intramolecular FRB–FKBP binding, which appends the molecular strain to the sandwiched luciferases in the probes. The control studies in Figure 1 collectively suggest that rapamycin mostly triggers intramolecular interactions, although a minor portion of the probes may form intermolecular binding or clusters.

The modeling in inset *a* shows that the molecular strain is appended to the sandwiched RLuc86SG*v3* in a way to expose the active site to the aqueous phase containing the substrate, although the initial active site is sterically hindered by FRB. However, the modeling in inset *b* indicates that the active site of ALuc49*v4* remains exposed from the outset, and the strain by rapamycin opens it further outwards to facilitate the access of the substrate in the aqueous phase. This illustration indicates that the FRB linked to the highly flexible N-terminal of ALuc49*v4* may disturb the substrate binding because of its flexibility or blocking the entrance of the substrate to the binding site. On the other hand, the formation of the FRB–FKBP complex triggered by rapamycin potentially improves the enzymatic stability of ALuc49 (reverse side of the binding site), leading to BL intensity boosting.

## 4. Conclusions

The present study demonstrates bright and highly sensitive single-chain molecular strain probe templates to visualize PPIs in mammalian cells. This template embeds full-length RLuc86SG or ALuc49 as reporter luciferases, and the utility was exemplified with the interaction between FRB and FKBP as a model PPI. The brightness and advanced S/B ratios were accomplished by a series of optimization of the template designs, including the truncation of the reporters and modification of the linkers. The optical properties of the template were featured in detail with the substrate-driven change in the BL spectra and in the S/B ratios. The putative substrate-binding chemistry and the working mechanisms of the templates were computationally modeled in the presence or absence of rapamycin. Considering that rapamycin is an important immunosuppressant, a possible anticancer therapeutic, and a widely used research tool [26], the templates with red-shifted bioluminescence recruitment can be utilized for various applications related to animal imaging and disease diagnosis models. The templates have potential to be applied in any PPI cases, with potentially high brightness and S/B ratios. Considering that these strain probe templates are applicable to various PPI models, the present study should broaden the scope of the bioassay toolbox, which harnesses the merits of luciferase reporters and the unique concept of the molecular strain probes into bioassays and molecular imaging.

## Data Availability

The data presented in this study are available on request from the corresponding author.

## Supplementary Materials

The following supporting information can be downloaded at: https://www.mdpi.com/article/10.3390/s23073498/s1, Supplementary Figure S1: The relative optical intensities and substrate specificity of selected marine luciferases in living mammalian cells. Supplementary Figure S2: Putative working mechanism of F-R86SG-F after stimulation of rapamycin and the structure of S6-bound RLuc86SG. Supplementary Figure S3: Putative working mechanism of F-A49*v4*-F triggered by rapamycin. Supplementary Figure S4: Visible and NIR BL images of microslides growing COS-7 cells containing F-R86SG*v3*-F or F-A49*v4*-F. Supplementary Figure S5: Absolute BL images of F-R86SG*v3*-F and F-A49*v4*-F in the presence or absence of rapamycin.
