# Supplementary material for: Bright Molecular Strain Probe Templates for Reporting Protein–Protein Interactions
Source: Sensors (Basel). 2023 Mar 27;23(7):3498. doi: 10.3390/s23073498 (PMC10098686; doi:10.3390/s23073498)
Supplement: Supplementary file 1 [file sensors-23-03498-s001.zip › sensors-2281289-supplementary.pdf]

sensors

Supplementary Information

Article

Bright Molecular Strain Probe Templates for Reporting Protein–Protein Interactions

Sung-Bae Kim [1,*], Tadaomi Furuta [2], Genta Kamiya [3], Nobuo Kitada [3], Ramasamy Paulmurugan [4] and Shojiro A. Maki [3]

[1] Environmental Management Research Institute (EMRI), National Institute of Advanced Industrial Science and Technology (AIST), Tsukuba 305-8569, Japan
[2] School of Life Science and Technology, Tokyo Institute of Technology, Yokohama 226-8501, Japan
[3] Department of Engineering Science, Graduate School of Informatics and Engineering, The University of Electro-Communications, Chofu 182-8585, Japan
[4] Molecular Imaging Program at Stanford, Bio-X Program, Stanford University School of Medicine, Palo Alto, CA 94304, USA
* Correspondence: kimu-sb@aist.go.jp

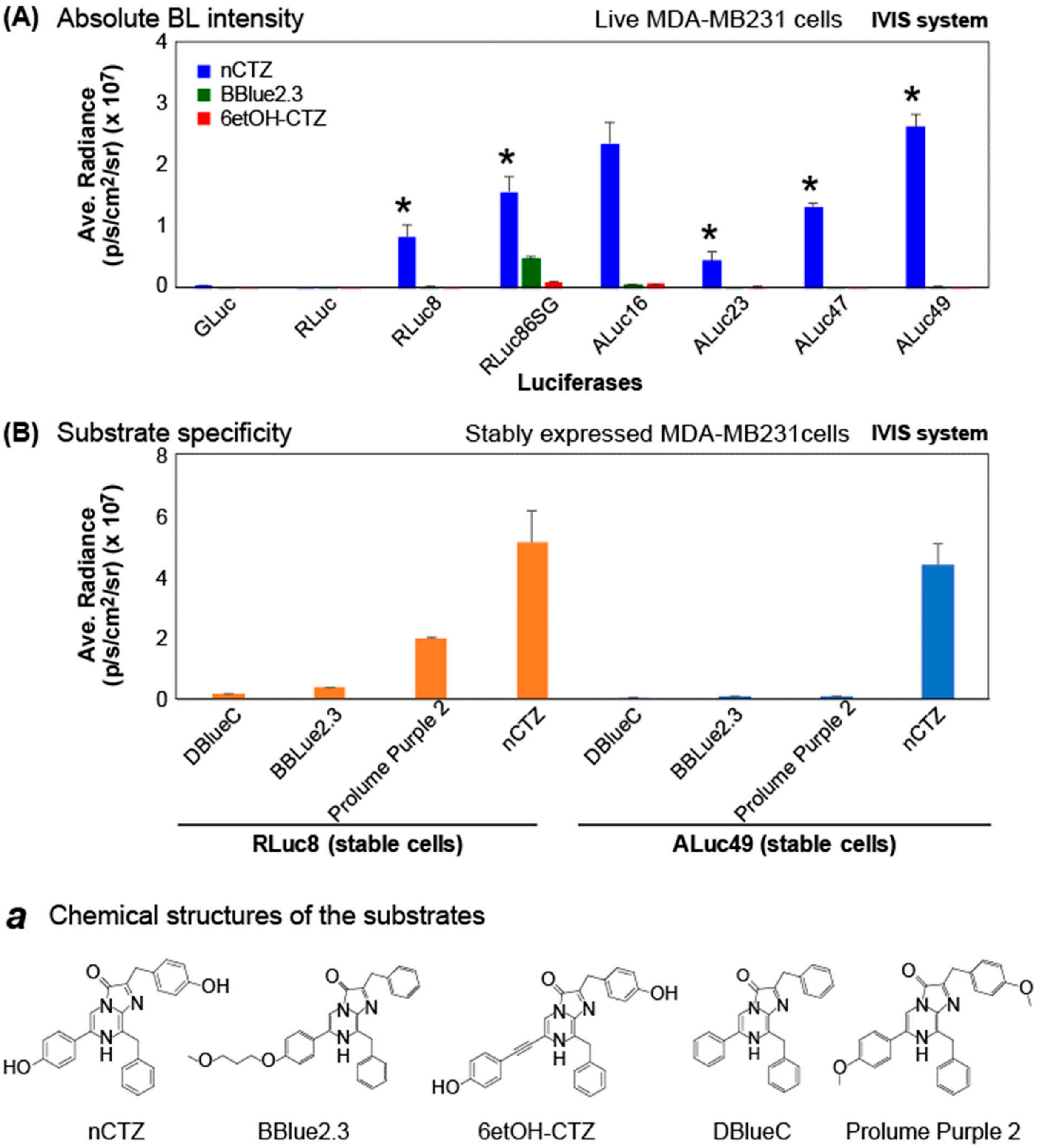

Supplementary Figure S1. (**A**) The relative optical intensities of selected marine luciferases, according to the substrates, in living mammalian cells. (**B**) Determination of the substrate specificity of RLuc8 and ALuc49. Inset *a* shows the chemical structures of the substrates used in this study.

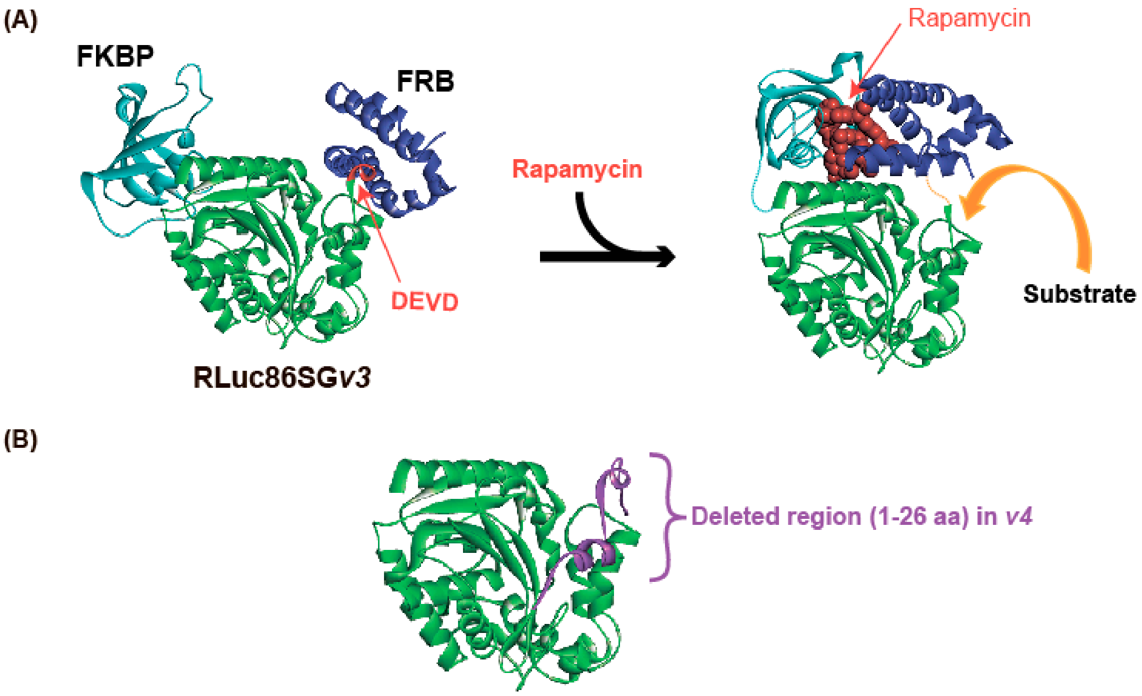

Supplementary Figure S2. Putative working mechanism of F-R86SG-F in the presence of rapamycin. (**A**) Putative BL mechanism of F-R86SG*v3*-F triggered by rapamycin. The RLuc8 and FKBP–FRB (with rapamycin) structures were obtained from the protein data bank (PDB: 1FAP 21 and 7OMR 20; the latter was modified to be RLuc86SG*v3*). These were manually deployed in the FRB-R86SG*v3*-FKBP order, similar to the previous study 16. In the FKBP–FRB bound state (right), the substrate can bind without the interference of the FRB. (**B**) The X-ray crystallographic information of RLuc86SG. The deleted region of RLuc86SG*v4* at the N-terminal was assigned with a false color of violet. The region (1–26 AA, purple) extends into the core (β sheet) of the enzyme, leading to the misfolding or attenuation of luminescence.

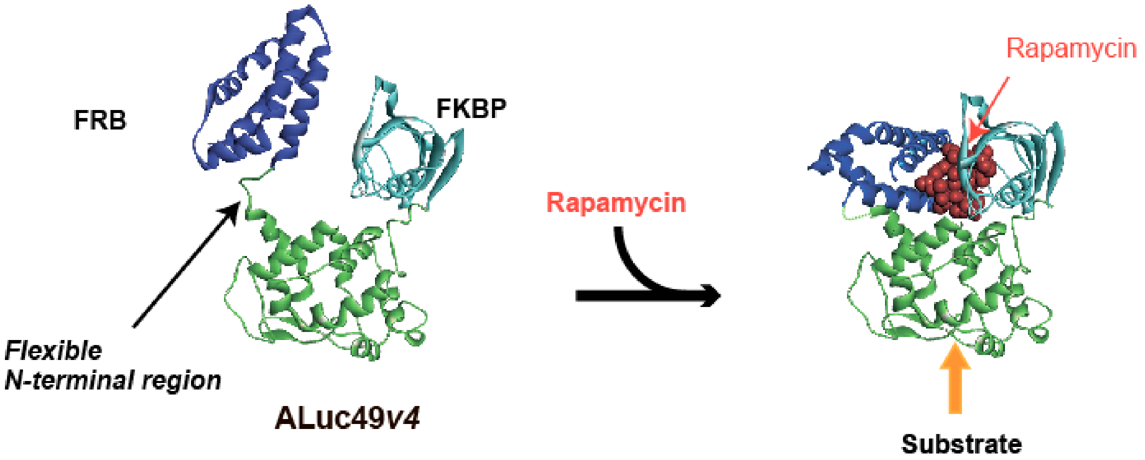

Supplementary Figure S3. Putative working mechanism of F-A49*v4*-F triggered by rapamycin. The ALuc49 structure was created by ColabFold 22 (then, the SP region was deleted to be ALuc49*v4*), and the FKBP–FRB structure used is the same in Suppl. Figure 2. These were manually deployed in the FRB-ALuc49*v4*-FKBP order. The FRB linked to the highly flexible N-terminal may disturb the substrate binding because of its flexibility or blocking the entrance of the binding site. On the other hand, the formation of the FRB–FKBP complex triggered by rapamycin may stabilize the backward region of ALuc49 (reverse side of the binding site), leading to BL restoration.

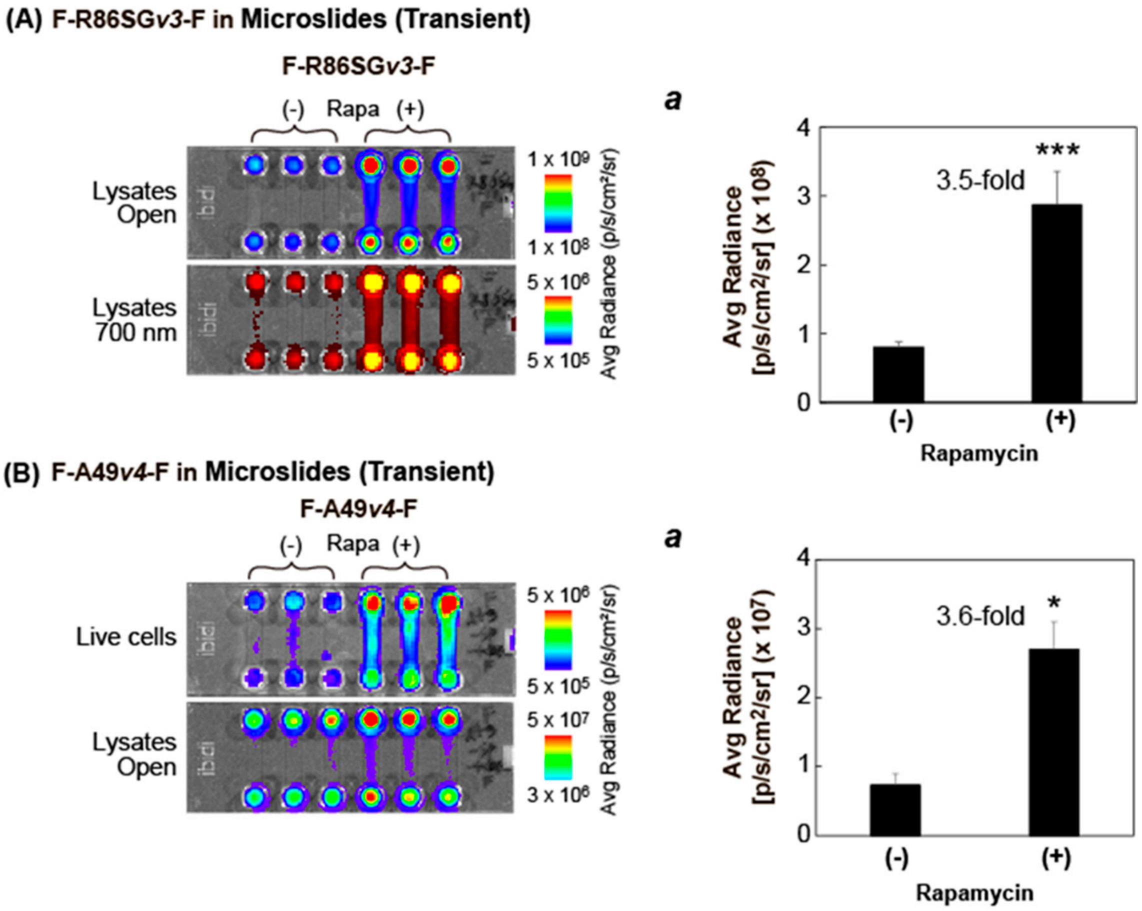

Supplementary Figure S4. (**A**) Visible and NIR BL images of COS-7 cells expressing F-R86SG*v3*-F grown in microslides (n = 3). The inset *a* shows the corresponding absolute BL intensities with and without rapamycin. The p-value (Student *t*-test) is *** < 0.001. (**B**) Visible BL images of COS-7 cells expressing F-A49*v4*-F grown in microslides (n = 3). The inset *a* shows the corresponding absolute BL intensities with and without rapamycin. The p-value (Student *t*-test) is * < 0.1.

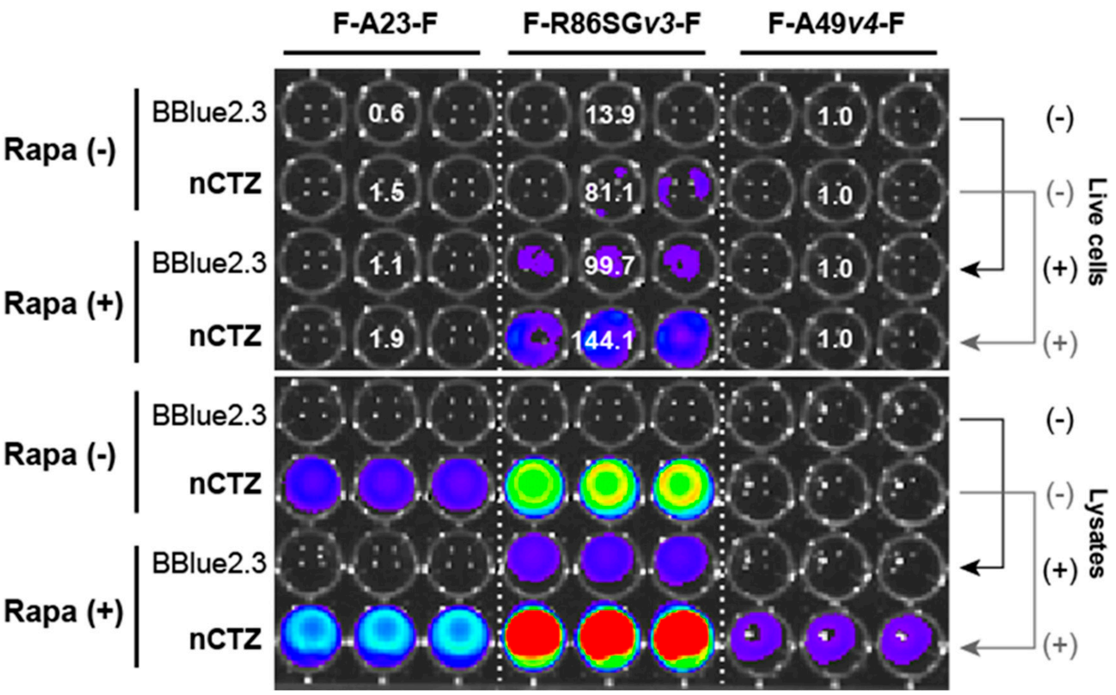

Supplementary Figure S5. BL image showing the absolute signal intensities of the selected molecular strain probes in the presence or absence of rapamycin (n = 3). The absolute BL intensities were developed with BBlue2.3 or nCTZ. The upper and lower panels are live COS-7 cells and the lysates, respectively. The numbers on the wells in the upper panel indicate the fold intensities of F-A23-F and F-R86SG*v3*-F, compared with that of F-A49*v4*-F in live COS-7 cells.
